# Institutional Readiness for Orofacial Laserology Education in Thailand: A National Cross-Sectional Survey of Dental Schools

**DOI:** 10.3390/dj14090559

**Published:** 2026-09-02

**Authors:** Suwat Tanya, Chattarika Domdok, Patcharawan Srisilapanan

**Affiliations:** 1 Department of Advanced General Dentistry and Dental Public Health, Faculty of Dentistry, Chiang Mai University, Chiang Mai 50200, Thailand; 2 Faculty of Dentistry, Chiang Mai University, Chiang Mai 50200, Thailand; chattarika.d@cmu.ac.th; 3 School of Dentistry, University of Phayao, Phayao 56000, Thailand

**Keywords:** orofacial laserology, dental education, institutional readiness

## Abstract

Background/Objectives: Orofacial laserology is being increasingly incorporated into contemporary dental practice, yet its integration into dental education remains inconsistent. National evidence regarding institutional readiness for integrating orofacial laserology into dental curricula remains limited. This study examined the current status of orofacial laserology education in Thailand. Methods: A national cross-sectional survey was conducted between May and July 2026 among all accredited Thai dental schools. Academic leaders and full-time lecturers completed a structured online questionnaire addressing curriculum implementation, educational delivery, competency assessment, institutional readiness, perceived barriers, and future priorities. Descriptive and non-parametric statistical analyses were performed. Results: Fifteen of 18 dental schools (83.3%) participated, yielding 38 respondents. Undergraduate orofacial laserology education showed limited curricular integration, whereas postgraduate programs demonstrated broader implementation, particularly in hands-on, supervised clinical, and competency-based training. Institutional readiness did not differ between academic leaders and full-time lecturers (*p* > 0.05). Across historical phases of institutional establishment, perceived importance (*p* = 0.743) and interinstitutional collaboration (*p* = 0.800) were comparable, whereas institutional readiness differed significantly (*p* = 0.008), with regional pioneer dental schools reporting higher readiness than expansion-phase and contemporary dental schools. The principal barriers were insufficient qualified instructors, limited curriculum integration, and high equipment costs. Faculty development, funding and equipment support, and national competency frameworks were identified as the highest priorities. Conclusions: Orofacial laserology education in Thailand remains heterogeneous, with broader implementation in postgraduate than undergraduate programs. Strengthening faculty capacity, competency-based curricula, institutional support, and national collaboration may facilitate more consistent integration of orofacial laserology into dental curricula.

## 1. Introduction

In this study, orofacial laserology describes an interdisciplinary field encompassing laser physics, laser–tissue interaction, laser safety, education, research, and clinical applications within the oral and maxillofacial region. Since the first reports of laser applications in 1965 [1], laser therapies have evolved from experimental techniques to clinically established modalities across multiple dental disciplines, including oral surgery, periodontology, endodontics, restorative dentistry, oral medicine, geriatric dentistry, special care dentistry and advanced general dentistry [2,3]. As laser technology becomes increasingly integrated into routine dental practice, dental graduates are expected to acquire the knowledge, clinical competencies, and safety awareness required for safe and effective use in patient care.

Formal education in orofacial laserology emerged in Europe and North America during the early 1990s and has subsequently expanded across undergraduate and postgraduate dental programs worldwide [1]. However, the integration into dental curricula has progressed unevenly. A survey of North American dental schools identified orofacial laserology as one of the most important emerging technologies in dentistry; nevertheless, only 39% of institutions reported clinical use of lasers for soft-tissue procedures and 12% for hard-tissue applications [4]. Similarly, although laser training within postgraduate periodontology programs increased from 76% in 2017 to 86% in 2022, educational activities frequently remained limited to brief didactic instruction and lacked supervised clinical experience [5,6]. These findings highlight the need for more comprehensive and competency-oriented approaches to orofacial laserology education.

Contemporary dental education increasingly emphasizes competency-based education, which integrates knowledge, psychomotor skills, clinical reasoning, professionalism, and patient safety [7]. Because laser applications require knowledge of laser–tissue interactions, laser safety, wavelength selection, and clinical decision-making, teaching orofacial laserology requires more than conventional lectures. Previous studies have shown considerable variation in curriculum integration, teaching methods, clinical training, and competency assessment [5,8,9,10]. Although many dental schools recognize the importance of laser education, implementation remains limited by insufficient hands-on learning, inadequate clinical exposure, and the lack of structured competency assessment. As a result, 90% of final-year dental students reported feeling unprepared to use lasers clinically, while 76% reported inadequate foundational knowledge of orofacial laserology [11,12]. Additional barriers, including limited faculty expertise, high equipment costs, and inadequate educational resources, continue to hinder curriculum integration.

Understanding institutional variation in laser education is particularly important because differences in curriculum structure, educational resources, faculty expertise, and clinical training opportunities may influence graduate preparedness and the safe adoption of laser technologies in clinical practice. Furthermore, because the expected scope of laser-related competencies differs between undergraduate and postgraduate training, evaluating both educational levels is necessary to understand how competency development is supported across the continuum of dental education.

Dental education in Thailand has evolved through three major phases (Table 1): the establishment of pioneering dental schools, the expansion of public institutions during the 1990s, and the rapid growth of public and private dental schools since 2004 [13]. This diverse educational landscape provides a valuable setting for examining how emerging technologies are integrated into dental curricula. Although orofacial laserology education has gradually expanded in Thailand, evidence describing its scope, structure, and implementation across dental schools remains limited. To date, no national study has comprehensively examined undergraduate and postgraduate orofacial laserology education in Thailand. Understanding the current educational landscape is essential for curriculum development, faculty capacity building, and the establishment of national competency standards. Therefore, this study aimed to examine the current status of undergraduate and postgraduate orofacial laserology education in Thailand, including curriculum implementation, educational resources, perceived barriers, and future priorities.

## 2. Materials and Methods

### 2.1. Study Design

This national cross-sectional survey was conducted between May and July 2026. This study was designed, conducted, and reported in accordance with the Strengthening the Reporting of Observational Studies in Epidemiology (STROBE) Statement for cross-sectional studies. Ethical approval was obtained from the Research Ethics Committee of the Faculty of Dentistry, Chiang Mai University (Approval No. 27/2026; approved on 21 April 2026). The study was conducted in accordance with the Declaration of Helsinki and institutional research ethics guidelines.

### 2.2. Study Sample

A census approach was adopted, in which all dental schools accredited by the Dental Council of Thailand and offering undergraduate and/or postgraduate dental programs during the study period were invited to participate. Because all eligible institutions were invited, no formal sample size calculation was performed. Each eligible institution was formally invited through an official invitation letter addressed to the dean, describing the study objectives, eligibility criteria, and instructions for completing the online questionnaire. Eligible participants were full-time lecturers with at least one year of teaching experience. Participants were classified into two groups: (1) academic leaders, including deans, associate or assistant deans for academic affairs, and program or course directors; and (2) faculty lecturers involved in teaching orofacial laserology or conducting research related to the field.

Because orofacial laserology is an emerging interdisciplinary field, educational responsibilities are often distributed across multiple departments and courses. Therefore, more than one respondent from each institution was invited to provide comprehensive institutional perspectives on curriculum implementation, educational resources, and clinical training.

### 2.3. Questionnaire Development

A structured online questionnaire was developed following a comprehensive review of the literature on orofacial laserology education and previous dental education surveys. The questionnaire was designed to collect respondent-level characteristics and institution-level information on undergraduate and postgraduate orofacial laserology education in Thailand. Before distribution, the questionnaire was reviewed independently by two experts in dental education and orofacial laserology to evaluate the relevance, clarity, comprehensiveness, and content validity. The questionnaire showed excellent content validity, with a scale-level content validity index based on the average approach (S-CVI/Ave) of 0.97 and a scale-level content validity index based on universal agreement (S-CVI/UA) of 0.95. A pilot test was then conducted with five eligible faculty members from three different universities to assess item clarity, questionnaire flow, completion time, and feasibility. As no changes to the questionnaire were required after the pilot test, the pilot participants were included in the final study sample.

The questionnaire consisted of five sections: (1) respondent and institutional characteristics; (2) undergraduate orofacial laserology education; (3) postgraduate orofacial laserology education; (4) faculty expertise and laser-related educational resources; and (5) future educational priorities and development needs (Supplementary File S1). The survey covered curriculum implementation, educational content, teaching methods, hands-on and clinical training opportunities, competency assessment, faculty expertise, laser-related resources, perceived barriers, institutional readiness, and future priorities. In this study, institutional readiness refers to the perceived preparedness of an institution to further develop orofacial laserology education. Most questions used categorical response options, while some allowed multiple responses, rating scales, or open-ended comments. Skip logic was used to display only relevant questions based on respondents’ previous answers, thereby reducing respondent burden and improving survey efficiency.

### 2.4. Data Collection

A Thai-language questionnaire was administered electronically through a web-based survey developed using Google Apps Script. Participation was voluntary and anonymous. Completion and submission of the questionnaire were considered to indicate informed consent. Reminder emails were sent periodically throughout the data collection period to maximize the institutional response rate. When responses were incomplete or inconsistent, respondents were contacted by email to verify or clarify the information before data analysis. When discrepancies in institution-level information were identified among multiple respondents from the same institution, clarification was obtained from the corresponding academic representative before finalizing the institutional dataset. Responses were exported into Microsoft Excel for data management prior to statistical analysis. To maintain confidentiality, survey responses were analyzed and reported in aggregate, and no institution-specific results were disclosed. No missing data remained after data verification.

### 2.5. Statistical Analyses

Data were analyzed using R version 4.5.3 (R Foundation for Statistical Computing, Vienna, Austria). Descriptive statistics were used to summarize survey responses. Categorical variables were summarized as frequencies and percentages, whereas ordinal variables were summarized using medians and interquartile ranges (IQRs), as appropriate. Differences in institutional readiness ratings among developmental phases were assessed using the Kruskal–Wallis test, followed by Holm-adjusted pairwise Wilcoxon rank-sum tests when the overall test was statistically significant. Comparisons between academic leaders and faculty lecturers were performed using the Wilcoxon rank-sum test. All statistical tests were two-sided, and a *p*-value < 0.05 was considered statistically significant.

## 3. Results

### 3.1. Institutional Participation and Respondent Characteristics

A total of 15 of the 18 dental schools accredited by the Dental Council of Thailand (83.3%) participated in the survey, yielding responses from 38 respondents, including academic leaders and full-time lecturers (Table 2). Participating institutions represented all three developmental phases of Thai dental education, providing broad perspectives on curriculum planning, educational implementation, faculty capacity, and institutional readiness for orofacial laserology education.

**Figure 1:**
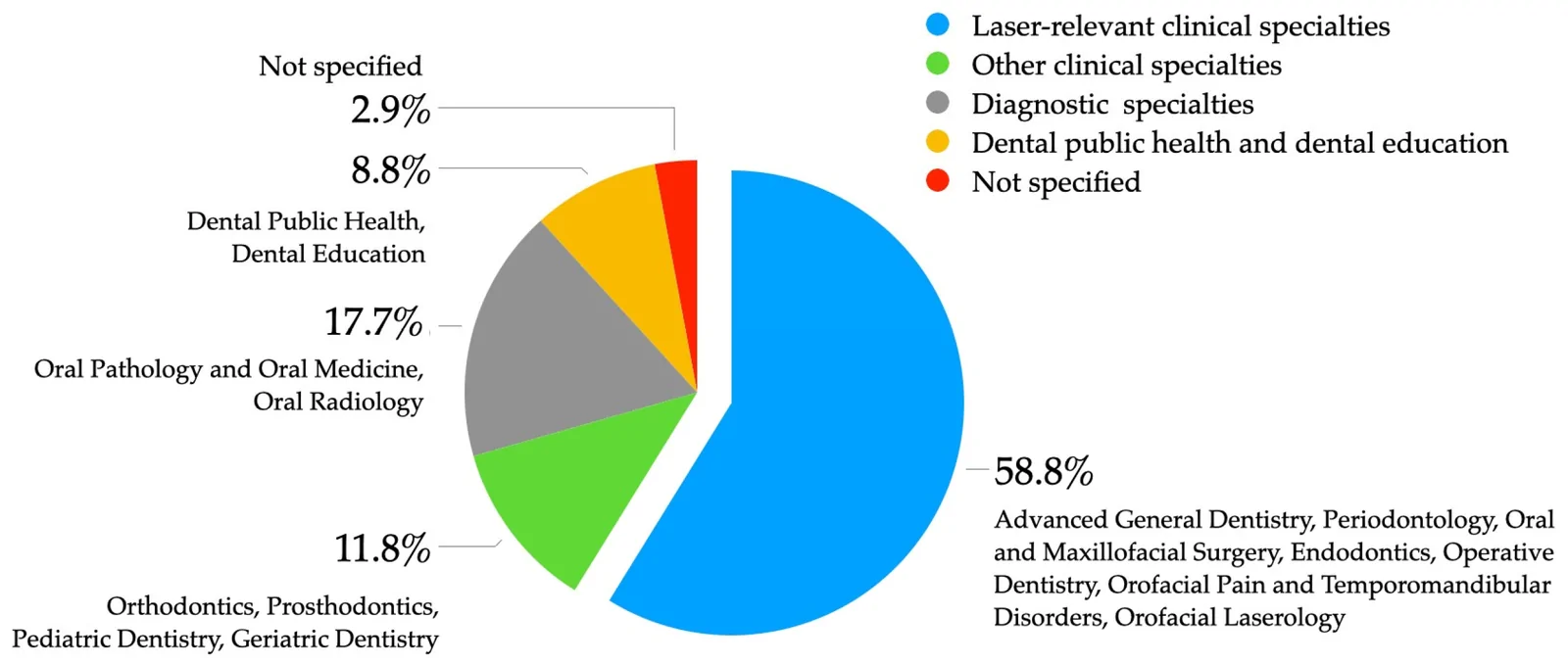
Distribution of respondents according to specialty group.

Respondents represented a wide range of dental disciplines (Figure 1). Individual specialties were grouped by the authors according to the relevance of routine clinical practice to orofacial laser applications. The largest category comprised laser-relevant clinical specialties (*n* = 20), followed by diagnostic specialties (*n* = 6), other clinical specialties (*n* = 4), and dental public health and dental education (*n* = 3); one respondent did not specify a specialty. Collectively, the respondent profile reflected multidisciplinary expertise from clinical, diagnostic, educational, and public health disciplines, supporting a comprehensive assessment of institutional perspectives on orofacial laserology education across Thai dental schools.

### 3.2. Current Implementation of Orofacial Laserology Education

Overall, implementation of orofacial laserology education varied considerably between undergraduate and postgraduate programs (Table 3 and Table 4). Undergraduate education demonstrated limited curricular integration, whereas postgraduate programs showed greater implementation, particularly in practical and clinical training. Notably, among all participating institutions, only one reported offering a dedicated orofacial laserology course, and this was confined to a postgraduate program. Across all developmental phases, most educational activities were delivered through integrated teaching within existing curricula. Practical training opportunities were concentrated primarily in postgraduate programs, while undergraduate education remained largely lecture-based with limited opportunities for preclinical simulation and supervised patient care.

Implementation patterns varied across the historical developmental phases of Thai dental schools (Table 3). Regional pioneer institutions demonstrated the broadest implementation of orofacial laserology education, including curriculum integration, comprehensive didactic content, and practical training opportunities. In contrast, implementation was minimal among expansion-phase institutions. Contemporary institutions showed initial incorporation of orofacial laserology into undergraduate curricula, particularly in the final year; however, dedicated educational activities and structured practical training remained limited. Educational delivery and assessment strategies also differed between undergraduate and postgraduate programs (Table 4). Faculty-delivered lectures and case-based discussions were the most frequently reported teaching methods, whereas active learning, continuing education activities, and competency-based assessment were reported more frequently in postgraduate programs.

**Figure 2:**
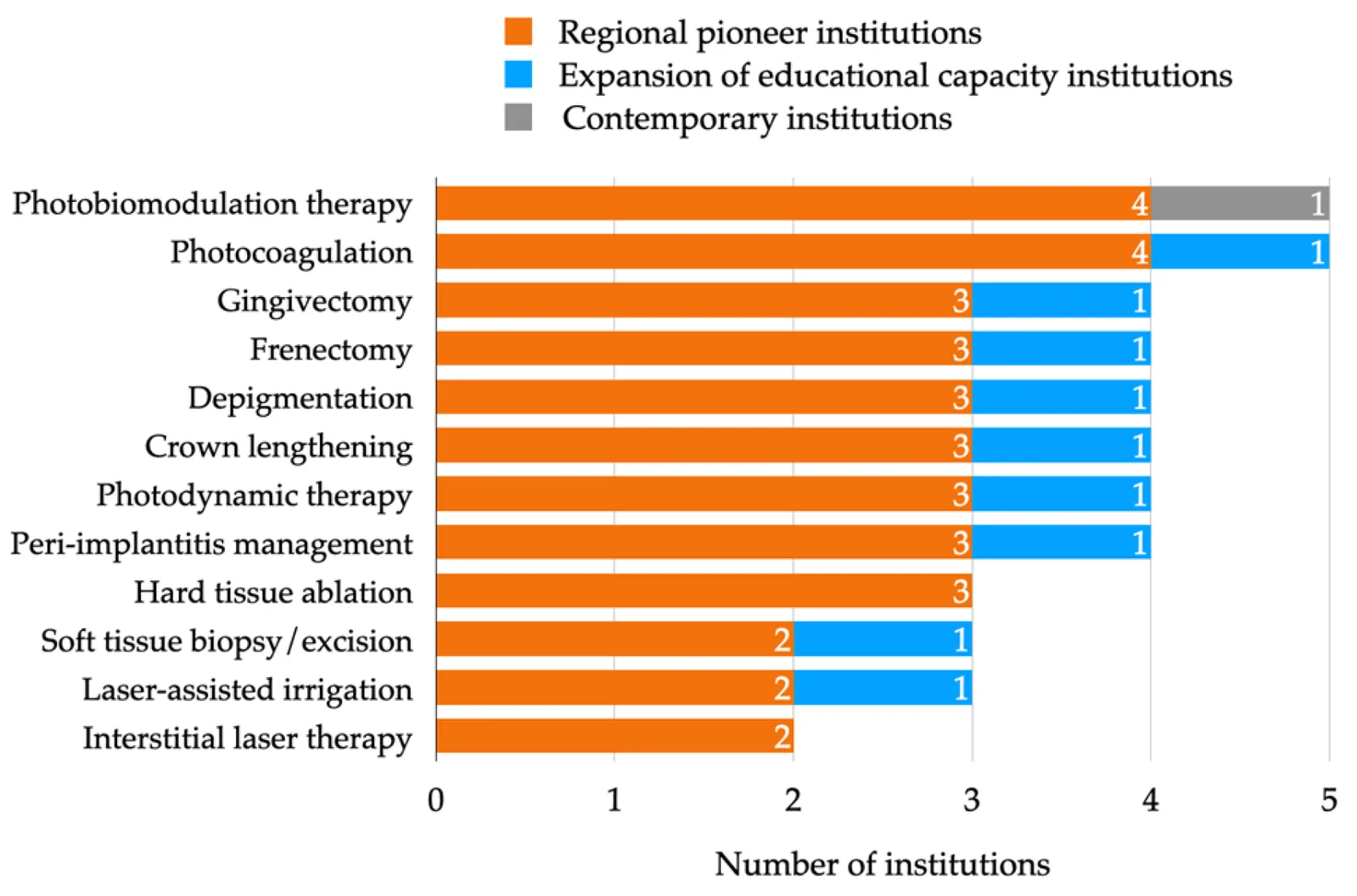
Availability of postgraduate hands-on laser procedures according to the historical phase of institutional establishment.

**Figure 3:**
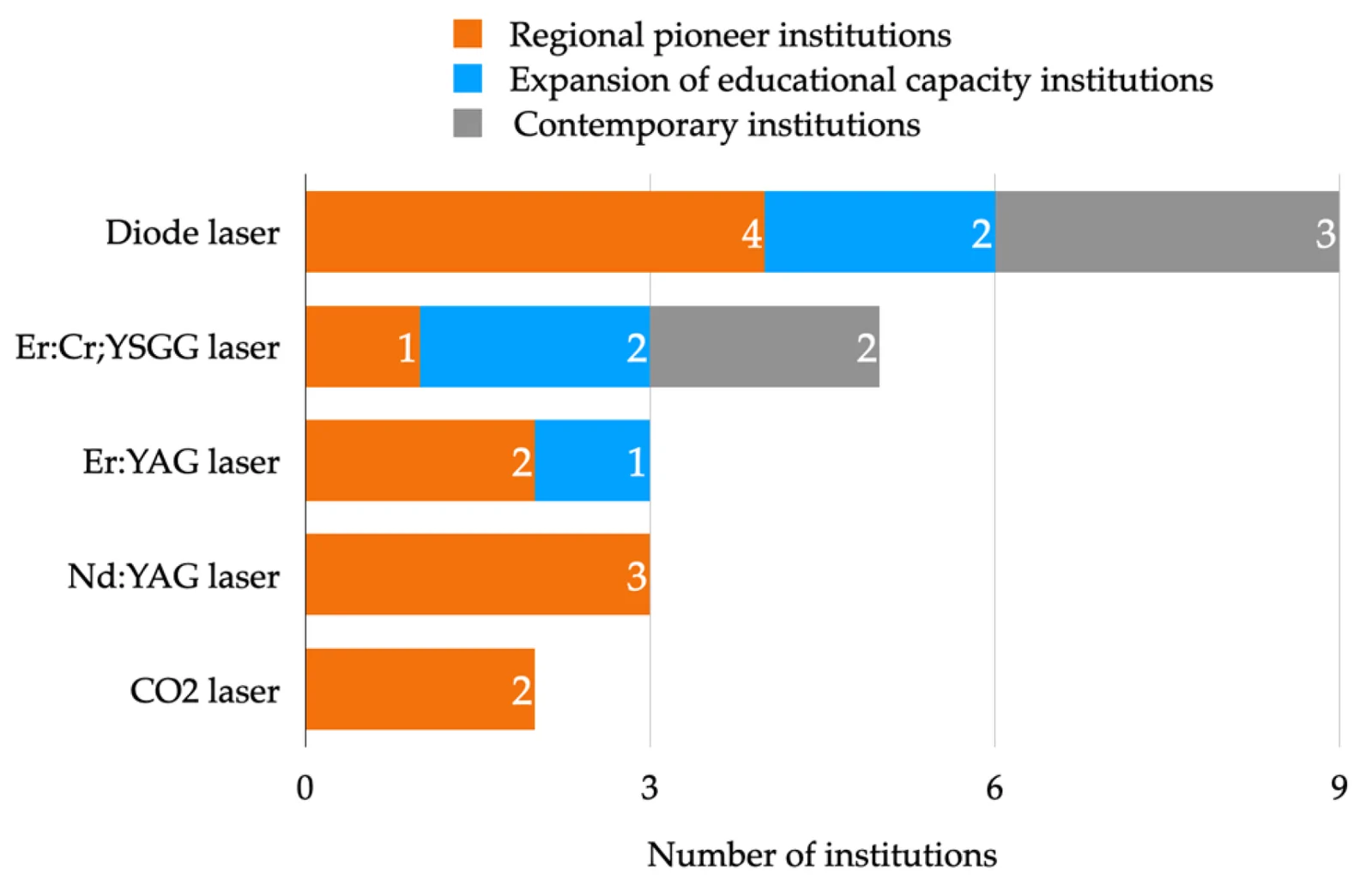
Availability of laser systems among participating Thai dental schools by historical phase of institutional establishment.

Unlike undergraduate programs, hands-on laser procedures were offered exclusively in postgraduate dental programs. These training opportunities were concentrated primarily in regional pioneer institutions, whereas expansion-phase and contemporary institutions provided a more limited range of hands-on laser procedures (Figure 2). The availability of laser systems also varied across the historical developmental phases of participating Thai dental schools (Figure 3). Diode lasers were the most widely available systems and were represented across all developmental phases. In contrast, Nd:YAG and CO_2_ lasers were available only in regional pioneer institutions, while Er:YAG systems were identified in regional pioneer and expansion-phase institutions. Er,Cr:YSGG lasers demonstrated the broadest distribution after diode lasers, extending across expansion-phase and contemporary institutions.

### 3.3. Institutional Readiness

**Figure 4:**
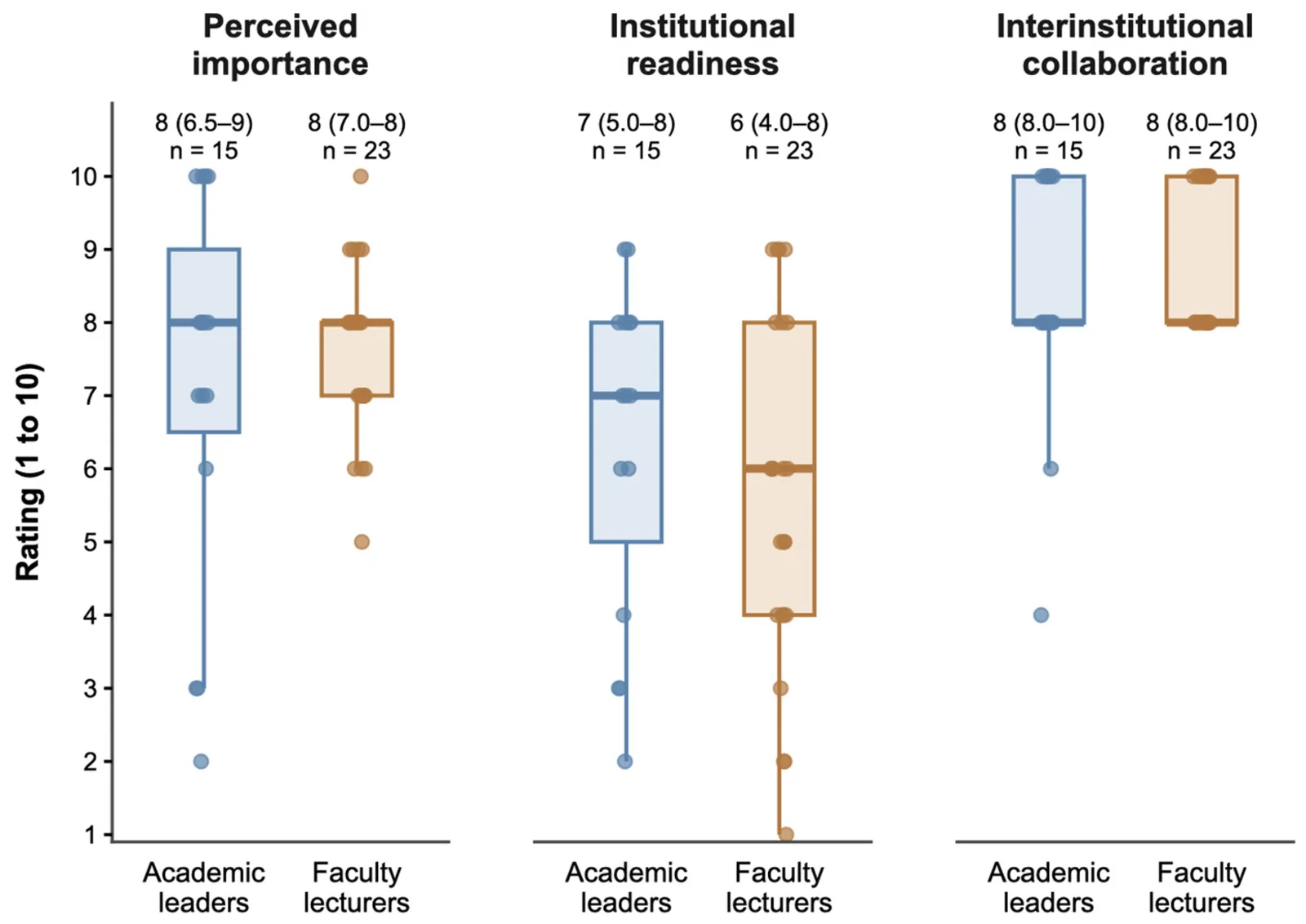
Comparison of institutional readiness ratings between academic leaders and lecturers. Values are presented as median (IQR).

**Figure 5:**
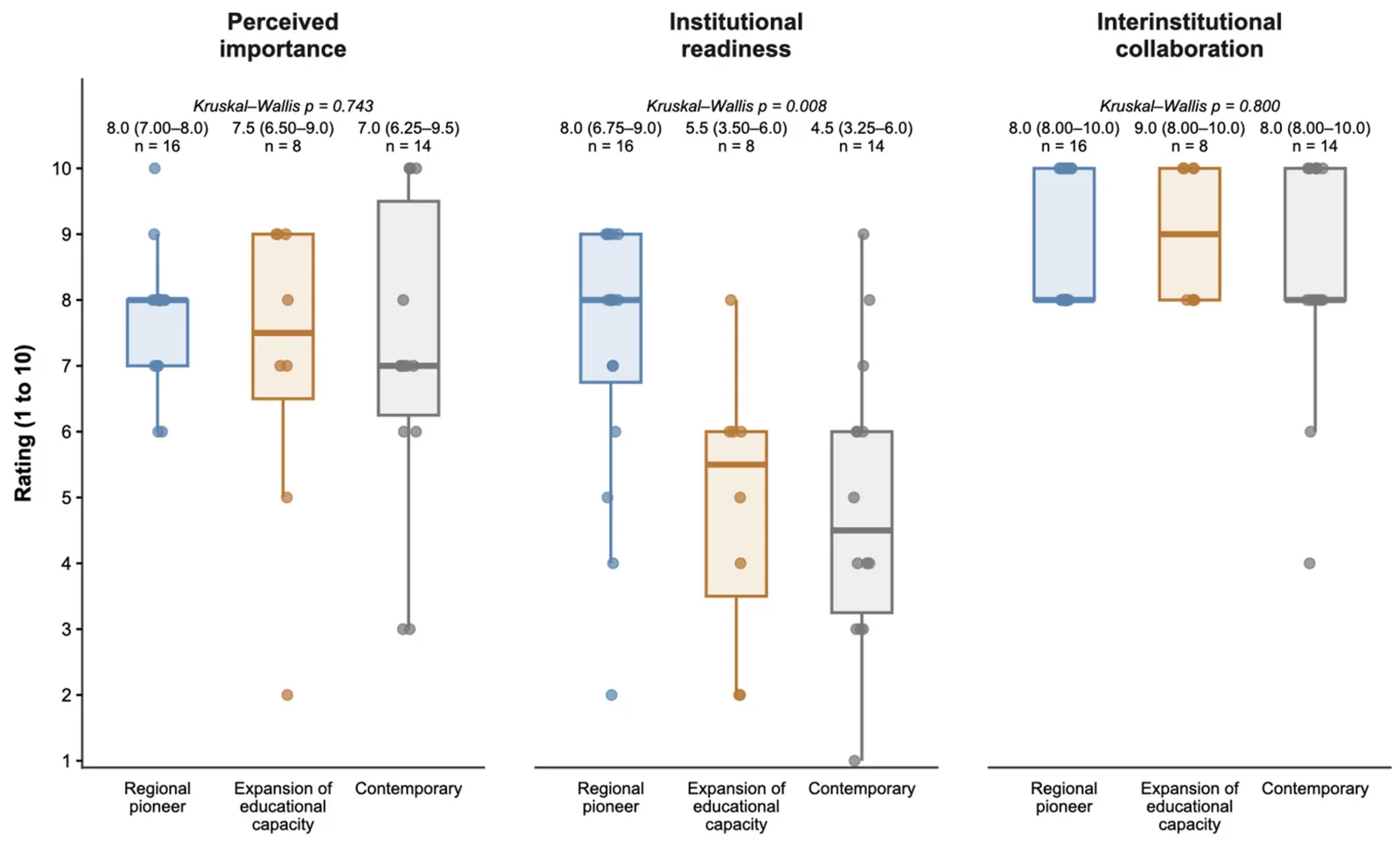
Comparison of institutional readiness ratings according to the historical developmental phases of Thai dental schools. Values are presented as median (IQR).

Ratings provided by academic leaders and faculty lecturers did not differ significantly across the three educational domains (Figure 4). No significant differences were observed between respondent groups in perceived importance (Wilcoxon rank-sum test, *p* = 1.000), institutional readiness (*p* = 0.382), or interinstitutional collaboration (*p* = 0.814). To examine institutional variation, ratings were subsequently compared across the historical developmental phases of Thai dental schools (Figure 5). Perceived importance of orofacial laserology education remained consistently high across all developmental phases, with no significant differences among regional pioneer, expansion-phase, and contemporary institutions (Kruskal–Wallis test, *p* = 0.743). Ratings for interinstitutional collaboration were likewise comparable across developmental phases (*p* = 0.800). In contrast, institutional readiness differed significantly across developmental phases (Kruskal–Wallis test, *p* = 0.008). Holm-adjusted pairwise comparisons showed that regional pioneer institutions reported significantly higher readiness than expansion-phase (*p* = 0.028) and contemporary institutions (*p* = 0.021), whereas no significant difference was observed between the latter two groups (*p* = 1.000).

### 3.4. Perceived Barriers to Implementation

**Figure 6:**
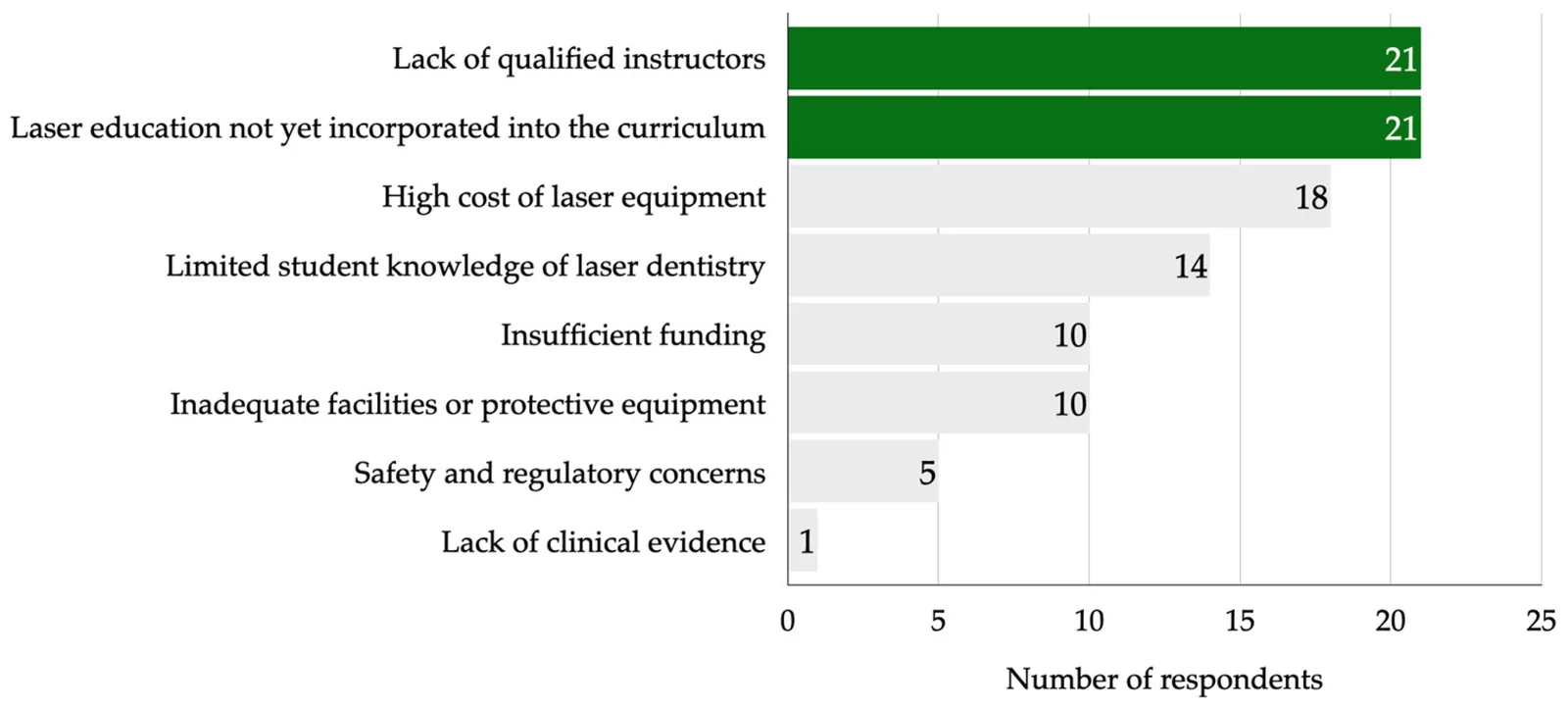
Perceived barriers to the implementation of orofacial laserology education. Multiple responses were permitted. The two most frequently reported barriers were highlighted in green.

The most frequently reported barriers to implementing orofacial laserology education were the lack of qualified instructors and the absence of orofacial laserology within existing curricula (Figure 6). Other commonly reported barriers included the high cost of laser equipment, limited student knowledge of laser dentistry, insufficient funding, and inadequate facilities or protective equipment. In contrast, safety and regulatory concerns and the lack of clinical evidence were reported less frequently.

### 3.5. National Priorities for Curriculum Development

**Figure 7:**
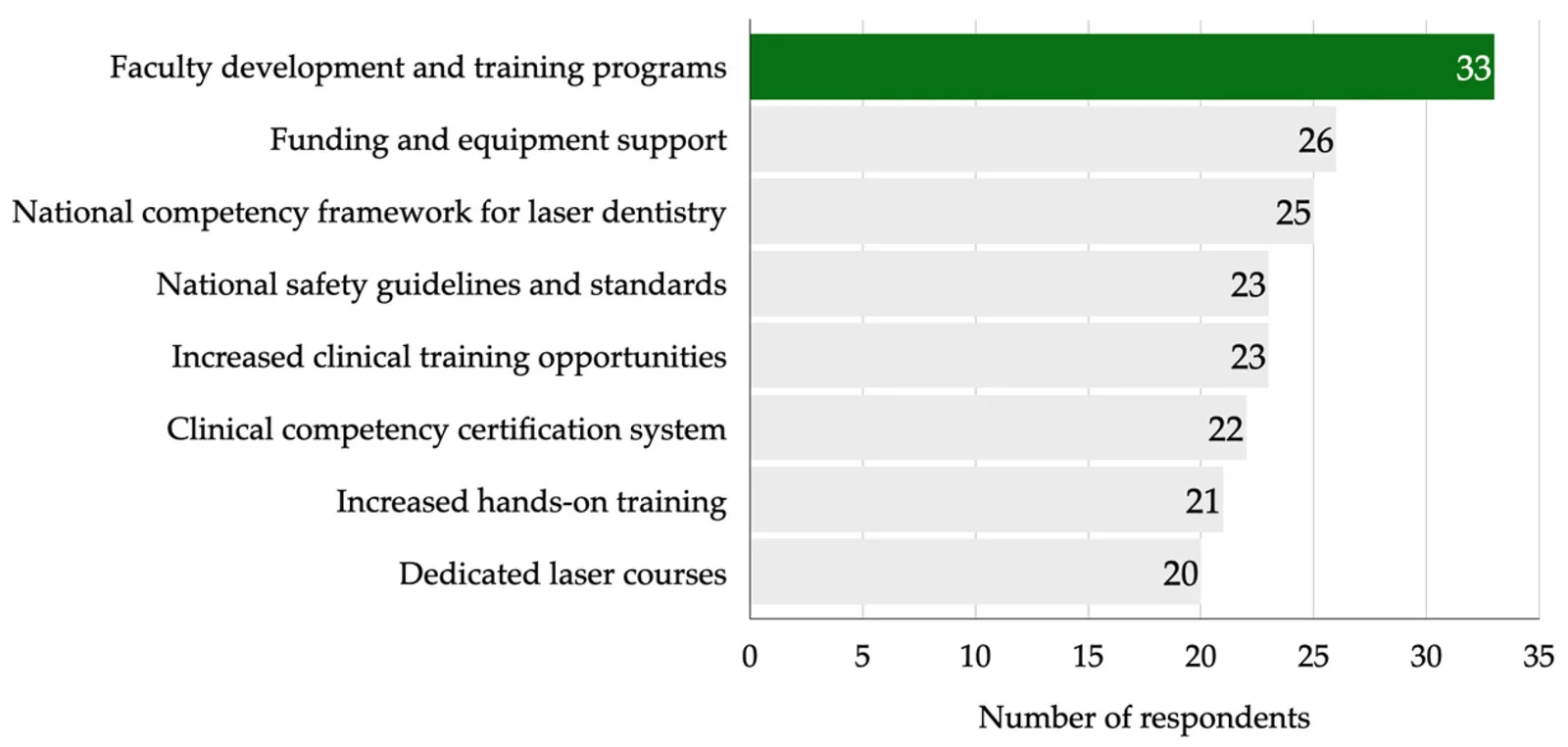
National priorities for advancing orofacial laserology education in Thai dental schools. Multiple responses were permitted. The highest-ranked national priority was highlighted in green.

Respondents identified faculty development and training as the highest national priority for advancing orofacial laserology education in Thailand (Figure 7). Other frequently identified priorities included funding and equipment support, the development of a national competency framework for laser dentistry, national safety guidelines and standards, increased clinical training opportunities, a clinical competency certification system, expanded hands-on training, and dedicated laser courses.

### 3.6. A Conceptual Competency Framework for Orofacial Laserology Education

**Figure 8:**
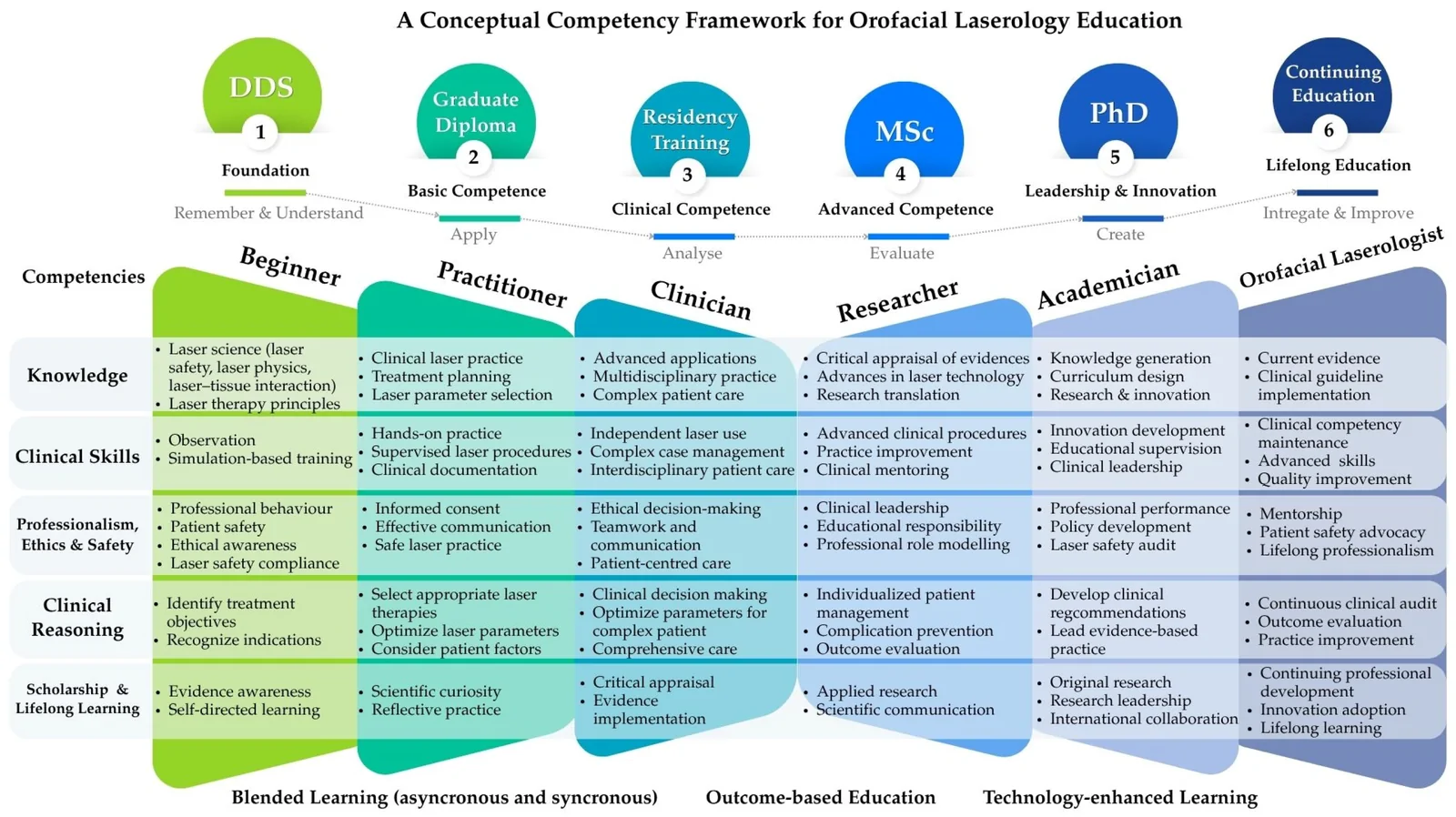
Proposed conceptual competency framework for orofacial laserology education. The upper row illustrates the educational continuum from undergraduate dental education to lifelong professional development, whereas the lower row depicts the corresponding progression of professional competency. Five core competency domains are integrated across all stages, with progressive cognitive development aligned with Bloom’s Revised Taxonomy.

Based on the findings of this national survey and informed by established competency-based educational principles [14], including the Thailand Qualifications Framework (TQF 2022) and Bloom’s Revised Taxonomy [15], we propose a conceptual competency framework for orofacial laserology education (Figure 8). The framework was developed by synthesizing the survey findings with relevant educational theories to illustrate a competency continuum spanning undergraduate dental education, postgraduate training, and lifelong professional development. It encompasses five core competency domains: knowledge, clinical skills, professionalism, ethics and safety, clinical reasoning, and scholarship with lifelong learning, while reflecting progressive cognitive development aligned with Bloom’s Revised Taxonomy. The framework also illustrates the progressive development of professional identity from beginner to orofacial laserologist across the educational continuum. Although conceptual and not intended as a validated competency model, the framework provides a practical reference for curriculum development, competency-based assessment, faculty development, and future national discussions on competency standards in orofacial laserology education. Further refinement and validation through structured expert consensus, such as the Delphi method, would be required before its formal adoption as a national competency framework.

## 4. Discussion

This survey suggests that the primary challenge for orofacial laserology education in Thailand is no longer whether laser technology is clinically relevant, but how it can be systematically integrated into dental curricula in a consistent and sustainable manner. As laser applications continue to expand across multiple dental specialties, dental schools need to prepare graduates with the knowledge, clinical skills, and professional competencies required for safe and appropriate laser use [1,16]. Similar challenges have been reported internationally, where the clinical adoption of laser technology has progressed faster than its integration into dental education [4,5,6]. Collectively, these findings suggest that Thailand faces challenges similar to those reported internationally and underscore the need for competency-based curriculum development that evolves alongside advances in clinical practice [14,17].

The differences between undergraduate and postgraduate laser education observed in this study are consistent with international experience. In many countries, laser education was initially established through specialist training and continuing professional development programmes before being progressively introduced into undergraduate curricula [1]. Consequently, postgraduate programmes generally provide greater opportunities for hands-on training and supervised clinical experience, whereas undergraduate education focuses on foundational knowledge, including laser safety, laser physics, laser–tissue interaction, and clinical indications [5]. Rather than introducing laser education as a stand-alone subject, our findings support its longitudinal integration throughout the dental curriculum [8]. Undergraduate education should emphasize foundational knowledge and clinical decision-making, while postgraduate programmes may progressively develop advanced clinical skills, research capability, and professional leadership. Such a staged approach allows competency development to progress in parallel with learners’ clinical experience while maintaining high levels of patient safety and educational quality [14,17,18].

Successful implementation of orofacial laserology education requires more than simply adding new teaching content [4,17]. It depends on qualified faculty, appropriate equipment, institutional support, and opportunities for practical learning [5,17]. Similar barriers have been reported in other countries, where limited faculty expertise, resource constraints, and competing curriculum priorities continue to delay curriculum integration [4,5]. Given that some institutions in this study reported industry-supported lectures or workshops, such collaboration may facilitate access to laser technologies and training resources, particularly in resource-limited settings. However, appropriate academic oversight is critical to ensuring that educational content remains evidence-based and independent of commercial influence, with transparency regarding potential conflicts of interest. Respondents consistently emphasized faculty development, national collaboration, and competency-based education rather than introducing a stand-alone laser course. They also advocated establishing a national knowledge hub to facilitate educational resource sharing, faculty development, collaborative research, and networking among dental schools. The observed differences in institutional readiness further highlight opportunities for inter-institutional collaboration. Institutions with established expertise and resources could serve as regional or national resource centers to support newer dental schools through shared faculty development, training resources, and collaborative education and research. In addition, respondents recommended a tiered curriculum that provides foundational competencies for all dental students while offering advanced clinical and research pathways for those seeking further specialization. Integrating laser education across multiple dental specialties was also considered important for promoting interdisciplinary learning and demonstrating the broad clinical applications of laser technology. Collectively, these findings suggest that embedding competency-based laser education within existing curricula may be more feasible than introducing a separate course, particularly for institutions with limited educational resources and faculty expertise [14,17,18].

Institutional readiness also appeared to vary according to the developmental phase of Thai dental schools. Institutions with longer experience in laser education tended to report greater faculty expertise, more established curriculum integration, and better educational resources than newer institutions [17]. These differences may reflect sustained investment in faculty development and educational infrastructure rather than differences in institutional priorities. The findings therefore suggest that curriculum implementation should be adapted to each institution’s level of readiness while maintaining shared national competency standards. Based on these findings, we propose a conceptual competency framework that links undergraduate education, postgraduate training, and lifelong professional development. Rather than prescribing a fixed curriculum, the framework provides a practical reference for curriculum planning, competency-based assessment, faculty development, and future national discussions on competency standards in orofacial laserology education.

This study is the first national survey of orofacial laserology education in Thailand and incorporates perspectives from academic leaders and faculty lecturers representing a broad range of dental disciplines. Participation from institutions across different developmental phases provides a comprehensive overview of the current educational landscape. Several limitations should be acknowledged. Although 15 of the 18 accredited Thai dental schools participated, the number of respondents per institution was limited. As respondents were purposively selected based on their institutional roles or involvement in orofacial laserology teaching or research, the findings reflect informed institutional perspectives rather than the views of all dental faculty members. Reliance on self-reported information may also introduce reporting bias, while potential non-response bias from the three non-participating institutions cannot be excluded. Furthermore, relatively few respondents were specialists in orofacial laserology, which may have influenced perspectives on laser education and curriculum implementation. Future studies involving orofacial laserology experts should further refine and validate the proposed competency framework through expert consensus before broader implementation. Overall, this study provides national baseline evidence to support curriculum development, faculty development, and educational policy for orofacial laserology in Thailand, while offering a practical reference for other countries seeking to integrate emerging technologies into competency-based dental education.

## 5. Conclusions

Orofacial laserology education in Thailand remains inconsistently integrated across dental curricula, with broader implementation in postgraduate than undergraduate education. Strengthening faculty capacity, competency-based education, institutional support, and national collaboration may facilitate more systematic integration. The proposed conceptual competency framework provides a foundation for future curriculum development and validation.

## Figures and Tables

**Table 1:** Classification of accredited Thai dental schools according to historical phase of institutional establishment.

Historical Phase of Institutional Establishment	Thai Dental School *	Established	Sector
National foundation and regional pioneer schools (1940–1983)	Faculty of Dentistry, Chulalongkorn University	1940	Public
	Faculty of Dentistry, Mahidol University	1968	Public
	Faculty of Dentistry, Chiang Mai University	1972	Public
	Faculty of Dentistry, Khon Kaen University	1979	Public
	Faculty of Dentistry, Prince of Songkla University	1983	Public
Expansion of educational capacity (1993–1999)	Faculty of Dentistry, Naresuan University	1993	Public
	Faculty of Dentistry, Srinakharinwirot University	1994	Public
	Faculty of Dentistry, Thammasat University	1996	Public
Contemporary expansion of dental education (2000 onward)	College of Dental Medicine, Rangsit University	2004	Private
	Faculty of Dentistry, Western University	2009	Private
	School of Dentistry, Mae Fah Luang University	2012	Public
	Faculty of Dentistry, University of Phayao	2012	Public
	Institute of Dentistry, Suranaree University of Technology	2015	Public
	International College of Dentistry, Walailak University	2016	Public
	Faculty of Dentistry, Bangkokthonburi University	2016	Private
	School of Dentistry, King Mongkut’s Institute of Technology Ladkrabang	2021	Public
	Faculty of Dentistry, Siam University	2023	Private
	Faculty of Dentistry, Burapha University	2024	Public

* Thai dental schools were identified based on accreditation by the Dental Council of Thailand as of 2026. Historical phases of institutional establishment were classified by the authors according to the year of establishment for analytical purposes.

**Table 2:** Characteristics of participating dental schools and respondents by historical phase of institutional establishment.

Phase of Thai Dental School Establishment	Institution-Level, *N*	Respondent-Level, *n*	
	Participating Institutions	Academic Leaders *	Full-Time Lecturers
Regional pioneer institutions (1940–1983)	5/5 (100%)	6	10
Expansion of educational capacity institutions (1993–1999)	3/3 (100%)	3	5
Contemporary expansion institutions (2000 onward)	7/10 (70%)	6	8
Total	15/18 (83.3%)	15	23

* Academic leaders included deans, associate deans, and program directors; *N*: number of institutions; *n*: number of respondents.

**Table 3:** Implementation of orofacial laserology education according to historical phase of institutional establishment.

Educational Component	Undergraduate Level			Postgraduate Level		
	Regional Pioneer (*N* = 5)	Expansion of Educational Capacity (*N* = 3)	Contemporary Expansion (*N* = 7)	Regional Pioneer (*N* = 5)	Expansion of Educational Capacity (*N* = 3)	Contemporary Expansion (*N* = 1)
Availability of didactic orofacial laserology education						
Dedicated orofacial laserology course	0	0	0	1	0	0
Orofacial laserology content integrated into other courses	4	0	3	3	1	0
Didactic contact hours						
1–2 h	2	0	1	0	0	0
3–5 h	2	0	2	3	1	0
>10 h	0	0	0	1	0	0
Didactic contents						
Laser safety and prevention	3	0	4	4	0	0
Laser physics	2	0	1	3	0	0
Laser–tissue interaction and applications across dental disciplines	3	0	3	4	1	0
Laser parameter setting	3	0	0	4	0	0
Practical training						
Preclinical hands-on training	0	0	1	4	1	0
Supervised clinical training with patients	2	0	0	4	1	0

*N*: number of institutions.

**Table 4:** Educational delivery and assessment strategies used in undergraduate and postgraduate orofacial laserology education in Thai dental schools.

Educational Delivery and Assessment Strategies Reported by Participating Institutions	Institution-Level, *N*	
	Undergraduate (*N* = 15)	Postgraduate (*N* = 9)
Laser safety requirements		
Mandatory laser safety training with competency assessment	4	2
No formal laser safety requirement	11	7
Teaching methods *		
Faculty-delivered lectures	7	6
Case-based discussions/seminars	4	5
Literature review/journal club	2	2
Research	2	2
E-learning/online modules	2	2
Industry-supported lectures/workshops	4	4
Active learning	1	3
Continuing education activities	0	3
Assessment methods *		
MCQ	7	3
Essay/written examination	3	2
Competency-based assessment	1	2
Self- and peer assessment	1	2
Logbook/clinical case requirements	1	3
OSCE	0	2

MCQ: Multiple-choice examination; OSCE: Objective Structured Clinical Examination; *N*: number of institutions; * Multiple responses were permitted.

## Data Availability

The datasets generated and analyzed during the current study are available from the corresponding author on reasonable request. Individual institutional responses are not publicly available because of institutional confidentiality restrictions.

## Supplementary Materials

The following supporting information can be downloaded at https://www.mdpi.com/article/10.3390/dj14090559/s1, Supplementary File S1: Questionnaire: National Survey of Orofacial Laserology Education in Undergraduate and Postgraduate Dental Curricula in Thailand.

## References

[1] Nammour S. (2016). Chronology of the Use of the Laser Beam in Dentistry, and the State of Postgraduate University Education Programs in This Domain. Photomed. Laser Surg..

[2] Grzech-Leśniak K. (2026). Lasers in Dentistry—Overview Based on FDI Policy Statement. Int. Dent. J..

[3] Tanya S., Srisilapanan P. (2026). Clinical Efficacy and Safety of Photobiomodulation Therapy for Orofacial Conditions in Older Adults: A Systematic Review of Randomized Controlled Trials. Dent. J..

[4] Brownstein S.A., Murad A., Hunt R.J. (2015). Implementation of New Technologies in U.S. Dental School Curricula. J. Dent. Educ..

[5] Hamada Y., Ricker A., Chiou L.-L., Prabhu S., Shin D.E., Blanchard S.B. (2022). Dental Laser Training and Education in Postgraduate Periodontics Programs in North America. J. Dent. Educ..

[6] da Silva A.P.B., Irfan U.M., Furman E., Jasinevicius T.R. (2022). Status of North American Graduate Programs in Periodontics Providing Laser Education and Clinical Training: A Cross-Sectional Survey. Dent. J..

[7] Chuenjitwongsa S., Oliver R.G., Bullock A.D. (2018). Competence, Competency-Based Education, and Undergraduate Dental Education: A Discussion Paper. Eur. J. Dent. Educ..

[8] Bordea R., Lucaciu O., Câmpian R.S. (2016). Student’s Knowledge and Opinion Regarding the Need of Implementation of Lasers in Dental Faculty Curriculum. Hum. Vet. Med..

[9] Gupta V., Mishra S., Sangha K.S., Rahman W., Goswami V., Thakkar V. (2022). Assessment of Knowledge and Practice of Lasers among Dental Professionals of Central India—An Online Questionnaire-Based Survey. J. Indian Assoc. Public Health Dent..

[10] Harini K., Arjunkumar R. (2018). Awareness of Laser Dentistry among Dentists in Tanjore—A Survey. Biomed. Pharmacol. J..

[11] Al-Jobair A. (2014). Dental Laser Education and Knowledge among Final Year Dental Students at King Saud University in Riyadh, Saudi Arabia. Saudi J. Dent. Res..

[12] Jayashree R.S., Kumar R.A. (2015). Dental Laser Education and Knowledge among Final Year Dental Students at Saveetha Dental College—A Questionnaire Study. Int. J. Adv. Res..

[13] Komabayashi T., Srisilapanan P., Korwanich N., Bird W.F. (2007). Education of Dentists in Thailand. Int. Dent. J..

[14] Frank J.R., Snell L.S., Ten Cate O., Holmboe E.S., Carraccio C., Swing S.R., Harris P., Glasgow N.J., Campbell C., Dath D. (2010). Competency-Based Medical Education: Theory to Practice. Med. Teach..

[15] Anderson L.W., Krathwohl D.R., Airasian P.W., Cruikshank K.A., Mayer R.E., Pintrich P.R., Raths J., Wittrock M.C. (2001). A Taxonomy for Learning, Teaching, and Assessing: A Revision of Bloom’s Taxonomy of Educational Objectives.

[16] Convissar R.A. (2015). Principles and Practice of Laser Dentistry.

[17] Gruppen L.D., Burkhardt J.C., Fitzgerald J.T., Funnell M., Haftel H.M., Lypson M.L., Mullan P.B., Santen S.A., Sheets K.J., Stalburg C.M. (2016). Competency-Based Education: Programme Design and Challenges to Implementation. Med. Educ..

[18] Frank J.R., Snell L., Englander R., Holmboe E.S., ICBME Collaborators (2017). Implementing Competency-Based Medical Education: Moving Forward. Med. Teach..

