# Supplementary material for: Institutional Readiness for Orofacial Laserology Education in Thailand: A National Cross-Sectional Survey of Dental Schools
Source: Dent J (Basel). 2026 Sep 2;14(9):559. doi: 10.3390/dj14090559 (PMC13605167; doi:10.3390/dj14090559)
Supplement: Supplementary file 1 [file dentistry-14-00559-s001.zip › dentistry-4506613-supplementary.pdf]

Questionnaire
National Survey of Orofacial Laserology Education in Undergraduate and Postgraduate Dental Curricula in Thailand

This study was approved by the Research Ethics Committee, Faculty of Dentistry, Chiang Mai University (Approval No. 27/2026).

Instructions

This questionnaire aims to investigate the current status of orofacial laserology education in undergraduate and postgraduate dental curricula in Thailand. The information collected will be used for research purposes only. Participation in this survey is voluntary. You may decline to answer any question or withdraw from the survey at any time without consequence. Completion and submission of the questionnaire will be considered as informed consent to participate in this study.

The questionnaire consists of five sections and requires approximately 5–7 minutes to complete.

1. Respondent and institutional characteristics
2. Undergraduate orofacial laserology education
3. Postgraduate orofacial laserology education
4. Faculty expertise and laser-related educational resources
5. Future priorities for orofacial laserology education in Thailand

Section 1. Respondent and Institutional Characteristics

1. Institution *(Select one)*

□ Chulalongkorn University □ Mahidol University □ Chiang Mai University
□ Khon Kaen University □ Prince of Songkla University □ Srinakharinwirot University
□ Thammasat University □ Naresuan University □ Rangsit University
□ Western University □ Mae Fah Luang University □ Walailak University
□ University of Phayao □ Siam University □ Burapha University
□ Suranaree University of Technology
□ Krungthepthonburi University
□ King Mongkut's Institute of Technology Ladkrabang

2. Respondent's role *(Select one)*

□ Dean □ Vice Dean / Assistant Dean □ Program Director
□ Full-time faculty member / Course instructor
□ Faculty member involved in orofacial laserology research
□ Other (please specify): __________________

3. Primary area(s) of expertise *(Select one)*

□ Oral Pathology and Oral Medicine □ Oral and Maxillofacial Radiology
□ Dental Public Health □ Advanced General Dentistry
□ Orthodontics □ Pediatric Dentistry
□ Endodontics □ Operative Dentistry
□ Crown and Bridge □ Periodontology
□ Prosthodontics □ Oral and Maxillofacial Surgery
□ Implant Dentistry □ Other (please specify): __________________

4. Dental program(s) offered at your institution *(Select all that apply)*

□ Doctor of Dental Surgery (DDS)
□ Postgraduate dental programs
□ Other (please specify): __________________

5. Approximate student enrollment

Annual undergraduate dental student enrollment ________ students/year
Annual postgraduate dental student enrollment________ students/year

Section 2. Undergraduate Orofacial Laserology Education

6. Does your Doctor of Dental Surgery (DDS) curriculum include didactic teaching in orofacial laserology? *(Select one)*

□ Yes, as a stand-alone course □ Yes, integrated into other courses
□ No *(Skip to Question 15)* □ Not sure *(Skip to Question 15)*

7. Approximately how many hours of didactic teaching in orofacial laserology are provided in the DDS curriculum? *(Select one)*

□ 1–2 hours □ 3–5 hours □ 6–10 hours □ More than 10 hours □ Not sure

8. Which teaching methods are used for didactic instruction in orofacial laserology? *(Select all that apply)*

□ Faculty-delivered lectures □ Case-based seminars/discussions
□ E-learning or online modules □ Literature review or journal club
□ Lectures or workshops delivered by laser manufacturers or distributors
□ Research activities □ Active learning
□ Continuing education (CE) activities □ Other (please specify): ___________________

9. Which topics are covered in the undergraduate orofacial laserology curriculum? *(Select all that apply)*

□ Laser safety and prevention □ Laser physics □ Laser–tissue interaction
□ Laser parameter setting □ Clinical applications of lasers across dental disciplines
□ Other (please specify): ___________________

10. Does the DDS curriculum include preclinical hands-on simulation training in orofacial laserology? *(Select one)*

□ Yes □ No

11. Which laser procedures are included in preclinical hands-on training? *(Select all that apply)*

□ Frenectomy □ Gingivectomy/Gingivoplasty
□ Photocoagulation □ Crown lengthening
□ Photobiomodulation therapy □ Soft tissue biopsy/excision
□ Hard tissue ablation □ Photodynamic therapy (PDT)
□ Interstitial laser therapy □ Laser-assisted irrigation
□ Peri-implantitis management □ Depigmentation
□ Other (please specify): ___________________

12. Are undergraduate dental students provided with supervised clinical training using lasers on patients? *(Select one)*

□ Yes □ No

13. Is formal laser safety training required before students are allowed to use dental lasers? *(Select one)*

□ Yes, with competency assessment □ Yes, without competency assessment
□ No formal requirement □ Not sure

14. How are students' competencies in orofacial laserology assessed? *(Select all that apply)*

□ Multiple-choice examination (MCQ) □ Written examination
□ Competency-based assessment □ Self- and peer assessment
□ Logbook or minimum clinical case requirements
□ Objective Structured Clinical Examination (OSCE)
□ Other (please specify): ___________________

Section 3. Postgraduate Orofacial Laserology Education

15. Does your postgraduate dental program include didactic teaching in orofacial laserology? *(Select one)*

□ Yes, as a stand-alone course □ Yes, integrated into other courses
□ No *(Skip to Question 25)* □ Not sure *(Skip to Question 25)*

16. Approximately how many hours of didactic teaching in orofacial laserology are provided in the postgraduate dental program? *(Select one)*

□ 1–2 hours □ 3–5 hours □ 6–10 hours □ More than 10 hours □ Not sure

17. Which teaching methods are used for didactic instruction in orofacial laserology?
(Select all that apply)

□ Faculty-delivered lectures □ Case-based seminars/discussions
□ E-learning or online modules □ Literature review or journal club
□ Lectures or workshops delivered by laser manufacturers or distributors
□ Research activities □ Active learning
□ Continuing education (CE) activities □ Other (please specify): ___________________

18. Which topics are covered in the postgraduate orofacial laserology curriculum?
(Select all that apply)

□ Laser safety and prevention □ Laser physics □ Laser–tissue interaction
□ Laser parameter setting □ Clinical applications of lasers across dental disciplines
□ Other (please specify): ___________________

19. Which postgraduate dental specialty programs include orofacial laserology education?
(Select all that apply)

□ Oral and Maxillofacial Surgery □ Periodontology □ Prosthodontics
□ Operative Dentistry □ Pediatric Dentistry □ Advanced General Dentistry
□ Oral Medicine □ Geriatric Dentistry □ Special Care Dentistry
□ Other (please specify): ___________________

20. Does the postgraduate dental program include preclinical hands-on simulation training in orofacial laserology? *(Select one)*

□ Yes □ No

21. Are postgraduate dental trainees provided with supervised clinical training using lasers on patients? *(Select one)*

□ Yes □ No

22. Which laser procedures are included in postgraduate hands-on training? *(Select all that apply)*

□ Frenectomy □ Gingivectomy/Gingivoplasty
□ Photocoagulation □ Crown lengthening
□ Photobiomodulation therapy □ Soft tissue biopsy/excision
□ Hard tissue ablation □ Photodynamic therapy (PDT)
□ Interstitial laser therapy □ Laser-assisted irrigation
□ Peri-implantitis management □ Depigmentation
□ Other (please specify): ___________________

23. Is formal laser safety training required before postgraduate trainees are allowed to use dental lasers?
(Select one)

□ Yes, with competency assessment □ Yes, without competency assessment
□ No formal requirement □ Not sure

24. How are postgraduate trainees' competencies in orofacial laserology assessed?
(Select all that apply)

□ Multiple-choice examination (MCQ) □ Written examination
□ Competency-based assessment □ Self- and peer assessment
□ Logbook or minimum clinical case requirements
□ Objective Structured Clinical Examination (OSCE)
□ Other (please specify): ___________________

Section 4. Faculty Expertise and Laser-related Educational Resources

25. How would you describe the availability of faculty members with expertise in orofacial laserology at your institution? *(Select one)*

□ Sufficient faculty expertise is available. □ Faculty expertise is available but insufficient.
□ No faculty member with expertise in orofacial laserology is available.
□ Not sure.

26. Does your institution have dental laser equipment available for teaching or hands-on training? *(Select one)*

□ Yes □ Yes, but the equipment is limited or insufficient
□ No *(Skip to Question 28)* □ Not sure *(Skip to Question 28)*

27. Which types of dental lasers are available for teaching or hands-on training at your institution? *(Select all that apply)*

□ Diode lasers
□ Carbon dioxide (CO_2) laser
□ Neodymium-doped yttrium aluminum garnet (Nd:YAG) laser
□ Erbium-doped yttrium aluminum garnet (Er:YAG) laser
□ Erbium, chromium-doped yttrium-scandium-gallium-garnet (Er,Cr:YSGG) laser
□ Other (please specify): ___________________

28. If your institution does not have sufficient dental laser equipment for teaching or hands-on training, what are the major barriers? *(Select all that apply)*

□ High cost of laser equipment
□ Insufficient budget
□ Lack of faculty members with expertise in orofacial laserology
□ Limited clinical evidence supporting laser applications
□ Safety and regulatory requirements
□ Orofacial laserology is not included in the curriculum
□ Inadequate facilities or safety equipment
□ Limited student demand or interest
□ Other (please specify): ___________________

Section 5. Future Priorities for Orofacial Laserology Education in Thailand

29. How important do you consider orofacial laserology education for the future Doctor of Dental Surgery (DDS) curriculum?

Rating scale (0 = Not important at all,10 = Extremely important)

□ 1 □ 2 □ 3 □ 4 □ 5 □ 6 □ 7 □ 8 □ 9 □ 10

30. In your opinion, how should orofacial laserology be incorporated into the undergraduate dental curriculum?

(Select one)

□ It is not necessary to include it in the curriculum.
□ It should be integrated into existing courses.
□ It should be included as a mandatory curriculum module.
□ It should be offered as a stand-alone course.
□ It should be offered as an elective course.
□ It should be developed as an online course for lifelong learning.
□ Not sure.

31. In your opinion, how should orofacial laserology be incorporated into postgraduate dental programs? *(Select one)*

□ It is not necessary to include it in the curriculum.
□ It should be integrated into existing courses.
□ It should be included as a mandatory curriculum module.
□ It should be offered as a stand-alone course.
□ It should be offered as an elective course.
□ It should be developed as an online course for lifelong learning.
□ Not sure.

32. How would you rate your institution's readiness to further develop orofacial laserology education?
Rating scale (0 = Not ready,10 = Fully prepared with a clear implementation plan)
□ 1 □ 2 □ 3 □ 4 □ 5 □ 6 □ 7 □ 8 □ 9 □ 10

33. Over the next five years, how do you expect orofacial laserology education at your institution to develop? *(Select all that apply)*
□ No plans for further development
□ Expand hands-on training
□ Develop a stand-alone course
□ Increase didactic teaching
□ Increase supervised clinical training
□ Not sure

34. What types of support would your institution need to further develop orofacial laserology education? *(Select all that apply)*
□ Funding or equipment
□ Faculty development and training
□ Laser safety guidelines and practice standards
□ Other (please specify): ___________________

35. In your opinion, which of the following initiatives would be most beneficial for advancing orofacial laserology education in Thailand? *(Select all that apply)*
□ National guidelines for orofacial laserology education
□ A competency framework for undergraduate and postgraduate dental education
□ A national certification system for clinical laser competency
□ Inter-institutional collaboration and networking
□ Other (please specify): ___________________

36. To what extent do you agree that collaboration among dental schools would strengthen orofacial laserology education in Thailand?
Rating scale (0 = Strongly disagree,10 = Strongly agree)
□ 1 □ 2 □ 3 □ 4 □ 5 □ 6 □ 7 □ 8 □ 9 □ 10

37. Please provide any additional comments or suggestions regarding the development of orofacial laserology education in Thailand. (openend question)
